# The prognostic role of HER2 expression in ductal breast carcinoma *in situ* (DCIS); a population-based cohort study

**DOI:** 10.1186/s12885-015-1479-3

**Published:** 2015-06-11

**Authors:** Signe Borgquist, Wenjing Zhou, Karin Jirström, Rose-Marie Amini, Thomas Sollie, Therese Sørlie, Carl Blomqvist, Salma Butt, Fredrik Wärnberg

**Affiliations:** 1 Division of Oncology and Pathology, Department of Clinical Sciences, Lund University, Medicon Village Building 404:B3, Scheelevägen 2, SE-223 81 Lund, Sweden; 2 Department of Surgical Science, Uppsala University, Uppsala, SE-75105 Sweden; 3 Department of Genetics and Pathology, Uppsala University, Uppsala, Sweden; 4 Department of Pathology, Örebro University, Örebro, Sweden; 5 Department of Genetics, Institute for Cancer Research, Oslo University Hospital, Norwegian Radium Hospital, Montebello, 0310 Oslo, Norway; 6 Department of Oncology, Helsinki University Central Hospital, Helsinki, Finland; 7 Department of Surgery, Clinical Sciences, Lund University, Malmö, Sweden

**Keywords:** Ductal carcinoma *in situ* (DCIS), HER2, Prognostic marker, Breast cancer

## Abstract

**Background:**

HER2 is a well-established prognostic and predictive factor in invasive breast cancer. The role of HER2 in ductal breast carcinoma *in situ* (DCIS) is debated and recent data have suggested that HER2 is mainly related to *in situ* recurrences. Our aim was to study HER2 as a prognostic factor in a large population based cohort of DCIS with long-term follow-up.

**Methods:**

All 458 patients diagnosed with a primary DCIS 1986–2004 in two Swedish counties were included. Silver-enhanced *in situ* hybridisation (SISH) was used for detection of HER2 gene amplification and protein expression was assessed by immunohistochemistry (IHC) in tissue microarrays. HER2 positivity was defined as amplified HER2 gene and/or HER2 3+ by IHC. HER2 status in relation to new ipsilateral events (IBE) and Invasive Breast Cancer Recurrences, local or distant (IBCR) was assessed by Kaplan-Meier survival analyses and Cox proportional hazards regression models.

**Results:**

Primary DCIS was screening-detected in 75.5 % of cases. Breast conserving surgery (BCS) was performed in 78.6 % of whom 44.0 % received postoperative radiotherapy. No patients received adjuvant endocrine- or chemotherapy. The majority of DCIS could be HER2 classified (*N* = 420 (91.7 %)); 132 HER2 positive (31 %) and 288 HER2 negative (69 %)). HER2 positivity was related to large tumor size (*P* = 0.002), high grade (*P* < 0.001) and ER- and PR negativity (*P* < 0.001 for both). During follow-up (mean 184 months), 106 IBCRs and 105 IBEs were identified among all 458 cases corresponding to 54 *in situ* and 51 invasive recurrences. Eighteen women died from breast cancer and another 114 had died from other causes. The risk of IBCR was statistically significantly lower subsequent to a HER2 positive DCIS compared to a HER2 negative DCIS, (Log-Rank *P* = 0.03, (HR) 0.60 (95 % CI 0.38–0.94)). Remarkably, the curves did not separate until after 10 years. In ER-stratified analyses, HER2 positive DCIS was associated with lower risk of IBCR among women with ER negative DCIS (Log-Rank *P* = 0.003), but not for women with ER positive DCIS.

**Conclusions:**

Improved prognostic tools for DCIS patients are warranted to tailor adjuvant therapy. Here, we demonstrate that HER2 positive disease in the primary DCIS is associated with lower risk of recurrent invasive breast cancer.

## Background

In general, patients with ductal breast carcinoma *in situ* (DCIS) have an excellent prognosis in terms of survival [1, 2]. However, the rate of new ipsilateral events (IBE) is high, even higher than following invasive breast cancer [2]. Almost half of all IBEs subsequent to a DCIS are invasive cancer and survival after an invasive IBE has, not surprisingly, been reported to be worse than after a non-invasive recurrence [3]. The knowledge about clinical and pathological predictors of recurrence following DCIS is currently limited [4, 5]. Certain sub-types of DCIS might be more prone to recur as invasive cancer and new biological markers may provide additional prognostic and/or treatment predictive value [6–9]. Recently, genomic-based data from a study using the Oncotype DX® indicated that it was possible to predict the local relapse risk for a primary DCIS independently from classical factors [10].

HER2 is an established negative prognostic factor in invasive breast cancer [11]. The prognostic significance of HER2 status in DCIS is, however, less clear [7, 12]. Both the relation of HER2 to risk of recurrence and its role in the progression from *in situ* to invasive cancer have been debated [12]. HER2 over-expression is reported to be more frequent in DCIS than in invasive cancer [13]. This may seem counterintuitive as HER2 is proposed to play a role in tumour progression. Some studies report an even higher proportion of HER2 positivity in micro-invasive cancer [14, 15] and, in pre-operative tumour biopsies displaying DCIS, HER2 over-expression has been related to a co-existing invasive component in the surgical specimen [16, 17]. Furthermore, HER2 is associated with high histopathological grade both in invasive cancer and in DCIS [18].

HER2 amplification is an important factor in the classification of molecular subtypes of invasive breast cancer [19]. An approximation of the gene expression based subtypes using immunohistochemical (IHC) detection of HER2, estrogen receptor- (ER) and progesterone receptor (PR) expression has been suggested for invasive breast cancer [20]. DCIS has been divided into the same surrogate molecular subtypes as invasive breast cancer [6, 21–23] but the prognostic value of these subgroups has not been convincing in DCIS [8, 24].

We have previously studied the surrogate molecular subgroups in this population-based DCIS cohort [8, 24]. Those studies indicated a lower risk of invasive recurrence in HER2-positive DCIS, although not statistical significant. We therefore conducted this updated analysis on the same patient population with further extended follow-up time and pooled HER2 data focusing on the role of HER2 status in DCIS and its relation to prognosis.

## Methods

### Patients

All patients diagnosed with a primary DCIS between 1986 and 2004 in two Swedish counties (Uppland and Västmanland) were included in this DCIS cohort (*n* = 458). The majority underwent breast-conserving surgery (79 %), and clear surgical margins were obtained in 88 % among all cases irrespective of surgical intervention. All cases were histopathologically re-examined. Tumour tissue biopsies of 1.0 mm in duplicate from paraffin blocks of samples taken as part of standard care were used to construct tissue microarrays (TMA). Follow up was complete up to December 15^th^, 2013. This study was approved by the Ethics Committee at Uppsala University, Sweden (Dnr 2005:118) and no informed consent was needed.

### Silver-enhanced *In Situ* Hybridization (SISH) and IHC

Scoring and IHC protocols are previously described in detail [21, 25]. SISH was performed on the automated instrument, Ventana Benchmark (Ventana Medical Systems, Tucson, AZ), as per the manufacturer’s protocols for the INFORM HER2 DNA probe and chromosome 17 probes. Testing for the HER2 gene and chromosome 17 was performed on sequential sections. Both probes are labelled with dinitrophenol and denaturation occurred on the instrument with enzyme digestion in protease 3 for eight minutes. The detection system used a multimer labelled with goat anti-rabbit antibody horseradish peroxidase as the linking step. Visualization occurred with the sequential addition of silver acetate as the source of ionic silver, hydroquinone, and hydrogen peroxide to give black metallic silver precipitate at the probe site. Counterstaining was performed with hematoxylin II on the instrument. The time taken for the complete run was 6.5 h. Both HER2 and chromosome 17-detection were performed on the same slide run. Gene amplification was assessed using the American Society of Clinical Oncology/College of American Pathologists guideline and Australian HER2 Advisory Board criteria for single HER2 probe testing (diploid, 1 to 2.5 copies/nucleus; polysemy >2.5 to 4 copies/nucleus; equivocal, >4 to 6 copies/nucleus; low-level amplification, >6 to 10 copies/ nucleus; and high-level amplification >10 copies/nucleus) and for dual HER2/CHR17 probe testing (non-amplified ratio <1.8; equivocal ratio, 1.8 to 2.2; gene amplification, >2.2) [26]. The HER2 status was predominantly relying on the SISH data. For those cases in which SISH failed, HER2 status was based on the IHC data and cases were considered HER2 positive if the IHC score was 3+, using the HercepTest©. As for hormone receptors (estrogen receptor alpha (ER) and progesterone receptor (PR)), tumours with 10 % or more nuclei stained were considered ER and PR positive, respectively. Staining intensity was not taken into consideration.

### Statistic analyses

Baseline characteristics among women with different HER2 status were compared by Chi-square test for categorical variables or analysis of variance for continuous variables. Survival analyses were performed using Kaplan-Meier curves, including the Log-Rank test. Cox proportional hazards regression models were used to generate hazard ratios (HRs) with 95 % confidence intervals (CIs), with adjustment for radiotherapy, age at diagnosis (continuous), tumour size (two categories: ≤ 25 mm; >25 mm or multifocal), and ER status. Stratification analyses were performed by mode of detection, type of surgery and ER in different multivariate models. All statistical tests were two-sided, and p-values less than 0.05 were considered significant. Data were analysed using the SPSS Statistics, version 19 (IBM, Chicago, IL, USA) and SAS 9.3 (Cary, NC, USA).

Primary endpoints were Ipsilateral Breast cancer Events (IBE) and Invasive Breast Cancer Recurrences (IBCR). IBEs were divided in new *in situ* events and invasive IBEs. As for IBCR, an event was defined as an invasive IBE, a regional recurrence, a contralateral invasive breast cancer or distant metastasis. IBCR free survival was calculated based on the time from primary surgery to the date of any invasive breast cancer event, date of death or date of last follow-up as by December 15^th^ 2013. For an event to be classified as IBE or IBCR, a minimum of 3 months had to pass after the primary surgery.

## Results

### Patient and tumour characteristics

Baseline characteristics by HER2 status are presented in Table 1. The mean age at diagnosis was 58.6 years, ranging from 30 to 90 years of age. A total of 202 women were diagnosed in Västerås, whereas the remaining 256 patients were diagnosed in Uppsala. In most cases, the DCIS was detected by mammographic screening (75.5 %). All patients except one underwent surgery with the majority of patients operated with breast conserving surgery (BCS) (78.6 %). Surgery was followed by postoperative radiotherapy (RT) in 35.2 % of all cases, although primarily for patients operated with BCS of whom 44.0 % received RT (Table 1). None of the patients received hormonal therapy or chemotherapy according to Swedish clinical guidelines.

**Table 1:** Baseline characteristics by HER2 status in 458 women with a primary DCIS

Characteristics	All	HER2 positive	HER2 negative		HER2 missing
	(*n* = 458)	(*n* = 132)	(*n* = 288)		(*n* = 38)
	n (%)	n (%)	n (%)	P-value^a^	n (%)
Age (years) mean (*n* = 458)	58.6	57.4	59.2	0.13	58.8
Detection mode (*n* = 457)					
Screening	345 (75.5 %)	108 (81.8 %)	210 (73.2 %)		27 (71.1 %)
Clinically	112 (24.5 %)	24 (18.2 %)	77 (26.8 %)	0.05	11 (28.9 %)
Type of Surgery (*n* = 457)					
BCS	359 (78.6 %)	98 (74.2 %)	233 (81.2 %)		28 (73.7 %)
Mastectomy	98 (21.4 %)	34 (25.8 %)	54 (18.8 %)	0.11	10 (26.3 %)
Radiotherapy (*n* = 359)					
Yes	158 (44.0 %)	49 (50.0 %)	100 (42.9 %)		9 (32.1 %)
No	201 (56.0 %)	49 (50.0 %)	133 (57.1 %)	0.32	19 (67.9 %)
Tumor size (mm) (*n* = 409)					
≤ 25	294 (71.9 %)	68 (61.3 %)	203 (77.2 %)		23 (65.7 %)
> 25 or multifocal	115 (28.1 %)	43 (38.7 %)	60 (22.8 %)	0.002	12 (34.3 %)
Nuclear grade (*n* = 458)					
Grade 1	42 (9.2 %)	1 (0.8 %)	34 (11.8 %)		7 (18.4 %)
Grade 2	176 (38.4 %)	20 (15.1 %)	145 (50.3 %)		11 (28.9 %)
Grade 3	240 (52.4 %)	111 (84.1 %)	109 (37.8 %)	<0.001	20 (52.6 %)
ER status (*n* = 419)					
Positive	307 (73.3 %)	59 (45.7 %)	243 (86.5 %)		5 (55.6 %)
Negative	112 (26.7 %)	70 (54.3 %)	38 (13.5 %)	<0.001	4 (44.4 %)
PR status (*n* = 409)					
Positive	213 (52.1 %)	35 (28.2 %)	175 (62.9 %)		4 (57.1 %)
Negative	196 (47.93 %)	89 (71.8 %)	103 (37.1 %)	<0.001	3 (42.9 %)

*BCS* breast conserving surgery, *RT* postoperative radiotherapy

^a^Comparisons between HER2-negative and HER2-positive DCIS were made by *χ*^2^-test, except for age and size that was compared by *T*-test

### Follow-up data

The mean follow up time was 183.5 months (range 3–329 months). A total of 105 IBEs were identified of which 54 were a new *in situ* and 51 an invasive IBE in the entire cohort of 458 patients. Among women treated with BCS (*N* = 324), recurrences were detected among 95 women comprising 49 *in situ* and 46 invasive cases. Eleven of the *in situ* IBEs were followed by a subsequent invasive IBE. One hundred and six IBCR were identified. The first invasive event was an invasive IBE in 50 cases, regional axillary metastasis in four, loco-regional together with distant metastases or distant metastasis only in twelve cases and a contralateral invasive cancer in 40 cases. In total, 32 patients developed generalized disease; fifteen subsequently to an invasive IBE, eight after an invasive contralateral cancer and nine had no reported prior local or regional recurrence. Eighteen women died from breast cancer and another 114 had died from other causes.

### HER2 status and patient – and tumor characteristics

Of all 458 women, 420 could be HER2 classified using available SISH or IHC data; 132 were classified as HER2 positive (31.4 %) and 288 as HER2 negative. Of 344 cases with available SISH data, 118 (34.3 %) were HER2 positive and of 408 cases with IHC data 103 (25.2 %) were classified as HER2 positive (i.e. 3+). In comparison, SISH and IHC data showed concordance in 296 of 332 (89.2 %) cases with available data for both SISH and IHC. Twenty-nine of those 332 cases were classified as HER2 2+ by IHC, 21 of those 29 (72.4 %) were SISH positive and eight (27.6 %) were SISH negative. In 38 cases, data on HER2 status was missing all together. HER2 status was significantly related to tumor size, nuclear grade (NG) and hormone receptor status, and the HER2 positive tumours tended to be detected by screening more often than HER2 negative tumors. HER2 status was not associated with age at diagnosis, type of surgery or RT (Table 1). In analyses assessing risk of recurrences according to established patient-and tumor characteristics, clinically detected DCIS were more prone to recur locally (HR 1.78 (1.04–3.07), larger DCIS lesions were correlated to a borderline significant increased risk of *in situ* IBEs but not invasive IBEs (HR 1.88 (0.97–3.62) and 0.64 (0.30–1.34)), respectively. Other assessed factors were not associated with recurrences (Table 2).

**Table 2:** Patient- and tumor characteristics of the primary DCIS in relation to risk of a breast cancer event. Risk of an ipsilateral breast cancer event (IBE) including risk of *in situ* IBEs and invasive ipsilateral recurrence, respectively, and any invasive recurrence (IBCR) (univariate Cox regression analyses)

Characteristics	Ipsilateral Breast Events (IBE)	*in situ* IBEs	*invasive* IBEs	Invasive Breast Cancer Recurrence (IBCR)
	Among BCS (*n* = 359)	BCS	BCS	Among all (*n* = 458)
	HR (95 % CI)	HR (95 % CI)	HR (95 % CI)	HR (95 % CI)
Age (years)				
< 50	Reference	Reference	Reference	Reference
50–65	0.77 (0.44–1.31)	0.92 (0.41–2.05)	0.63 (0.30–1.32)	0.68 (0.41–1.12)
> 65	1.19 (0.67–2.12)	1.31 (0.55–3.11)	1.09 (0.50–2.37)	0.98 (0.57–1.66)
Detection mode				
Screening	Reference	Reference	Reference	Reference
Clinically	1.78 (1.04–3.07)	1.78 (0.81–3.91)	2.09 (0.98–4.42)	1.47 (0.93–2.35)
Type of Surgery				
BCS	-	-	-	Reference
Mastectomy	-	-	-	0.55 (0.29–1.03)
RT after BCS				
No	Reference	Reference	Reference	Reference
Yes	0.53 (0.33–0.86)	0.50 (0.25–1.01)	0.51 (0.27–0.98)	0.91 (0.59–1.41)
Tumor size (mm)				
≤ 25	Reference	Reference	Reference	Reference
> 25 / multifocal	1.14 (0.71–1.83)	1.88 (0.97–3.62)	0.64 (0.30–1.34)	0.89 (0.55–1.45)
Nuclear grade				
Grade 1	Reference	Reference	Reference	Reference
Grade 2	0.90 (0.41–1.96)	1.05 (0.30–3.63)	0.73 (0.27–1.99)	0.82 (0.38–1.77)
Grade 3	0.83 (0.39–1.79)	1.03 (0.30–3.49)	0.63 (0.23–1.69)	0.64 (0.30–1.37)
ER status				
Negative	Reference	Reference	Reference	Reference
Positive	0.90 (0.52–1.55)	0.80 (0.38–1.71)	1.02 (0.47–2.22)	1.16 (0.70–1.93)
PR status				
Negative	Reference	Reference	Reference	Reference
Positive	0.83 (0.52–1.32)	1.04 (0.53–2.02)	0.66 (0.35–1.27)	1.06 (0.69–1.63)

*RT* radiotherapy, *BCS* breast conserving surgery, *IBE* ipsilateral breast events, *IBCR* invasive breast cancer recurrence

### HER2 and survival analyses

**Fig. 1:**
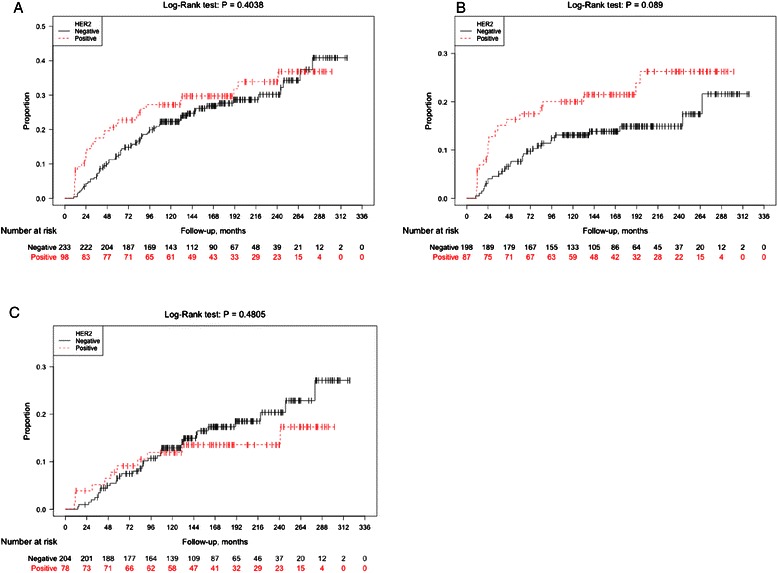
Ipsilateral Breast cancer Events (IBE) according to HER2 status of the primary DCIS. Kaplan-Meier plots showing ipsilateral recurrence-free survival analyses (IBE) among women with DCIS treated with breast conserving surgery (BCS) with respect to HER2 status of the primary DCIS regarding all ipsilateral events (**a**), ipsilateral *in situ* events (**b**), and ipsilateral invasive events (**c**)

Among the 420 women with available HER2 status, a total of 102 women experienced recurrences during follow-up. HER2 positivity in the primary DCIS was not a risk factor for IBE in women undergoing BCS (Log-Rank *P* = 0.40, HR 1.20 (95 % CI, 0.78–1.85)), (Fig. 1a and Table 3). Interestingly, divided by type of IBE, HER2 positivity showed a borderline statistically significant increased risk of *in situ* IBEs (Log-Rank *P* = 0.09, HR 1.63 (95 % CI, 0.92–2.89), and no association with risk of invasive IBEs (Log-Rank *p* = 0.48, HR 0.78 (95 % CI, 0.40–1.55)), (Fig. 1b–c and Table 3). Results from the multivariate analyses did not differ substantially from the univariate analyses (Table 3).

**Table 3:** Cox regression analyses by HER2 status in 458 women with a primary DCIS

	DCIS	
	HER2 positive	HER2 negative
	HR (95 % CI)	HR (95 % CI)
Ipsilateral Breast Events (IBE)		
BCS (*n* = 331) (events = 95)		
Univariate HR (95 % CI)	1.20 (0.78–1.85)	(Ref)
^a^Adjusted HR (95 % CI)	1.27 (0.83–1.96)	(Ref)
^b^Adjusted HR (95 % CI)	1.17 (0.75–1.83)	(Ref)
^c^Adjusted HR (95 % CI)	1.22 (0.76–1.95)	(Ref)
BCS, *in situ* IBEs (events = 49)		
Univariate HR (95 % CI)	1.63 (0.92–2.89)	(Ref)
^a^Adjusted HR (95 % CI)	1.76 (0.99–3.11)	(Ref)
^b^Adjusted HR (95 % CI)	1.40 (0.77–2.54)	(Ref)
^c^Adjusted HR (95 % CI)	1.65 (0.88–3.11)	(Ref)
BCS, invasive IBEs (events = 46)		
Univariate HR (95 % CI)	0.78 (0.40–1.55)	(Ref)
^a^Adjusted HR (95 % CI)	0.83 (0.42–1.64)	(Ref)
^b^Adjusted HR (95 % CI)	0.87 (0.43–1.75)	(Ref)
^c^Adjusted HR (95 % CI)	0.80 (0.38–1.66)	(Ref)
Invasive Breast Cancer Recurrence (IBCR)		
All patients (*n* = 420) (Events = 102)		
Univariate HR (95 % CI)	0.60 (0.38–0.94)	(Ref)
^a^Adjusted HR (95 % CI)	0.58 (0.38–0.95)	(Ref)
^b^Adjusted HR (95 % CI)	0.59 (0.37–0.95)	(Ref)
^c^Adjusted HR (95 % CI)	0.59 (0.36–0.98)	(Ref)

*BCS* breast conserving surgery

^a^Adjusted for radiotherapy

^b^Adjusted for radiotherapy, age at diagnosis (continuous) and size

^c^Adjusted for radiotherapy, age at diagnosis and ER

**Fig. 2:**
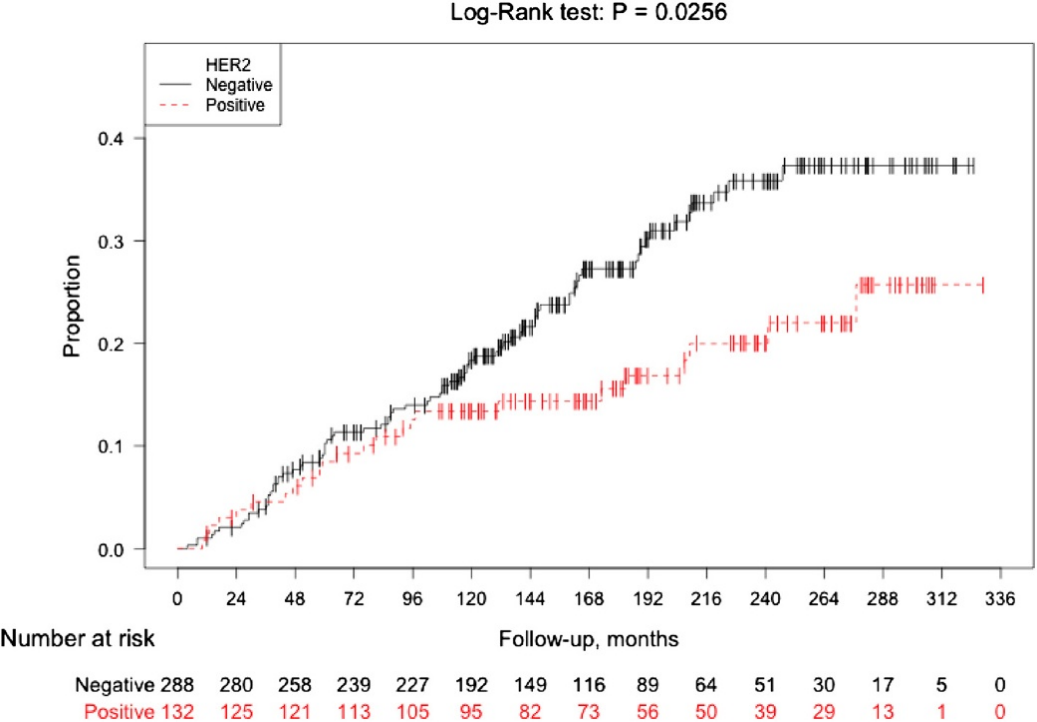
Invasive recurrence-free-survival according to HER2 status of the primary DCIS. Kaplan-Meier plot showing invasive recurrence-free survival analyses (IBCR) among women with a DCIS with respect to HER2 status of the primary DCIS

The risk of IBCRs was statistically significantly lower subsequent to a HER2 positive DCIS compared to a HER2 negative primary DCIS (Log-Rank *P* = 0.03, HR 0.60 (95 % CI, 0.38–0.94)) (Fig. 2 and Table 3). Remarkably, the curves in the Kaplan-Meier plot did not separate until after almost 10 years (Fig. 2). In the multivariate analyses, HRs after adjustments were very similar to the crude analyses (Table 3).

**Fig. 3:**
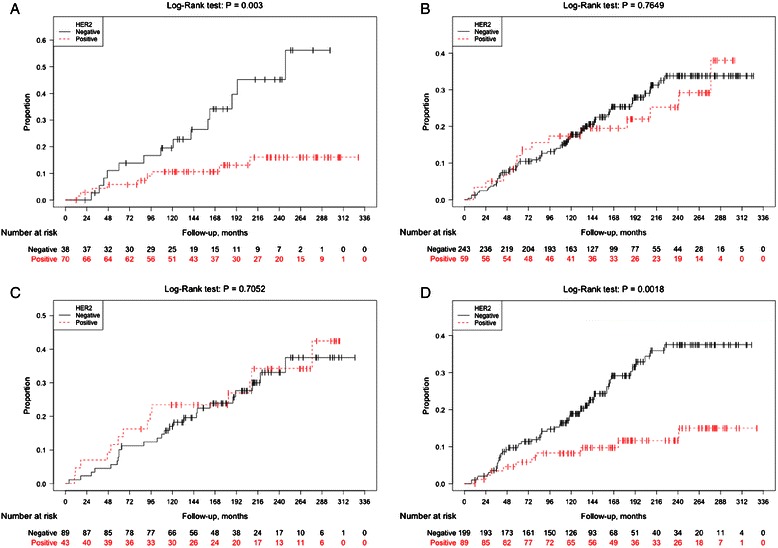
Invasive recurrence-free-survival according to HER2 of the primary DCIS, stratified for ER status and age, respectively. Kaplan-Meier analyses stratified for ER status of the primary DCIS, demonstrating invasive recurrence-free survival (IBCR) with respect to HER2 status of the primary DCIS among ER negative (**a**) and ER positive DCIS patients (**b**). Additional Kaplan-Meier curves show analyses stratified for age at diagnosis (≤50 years/> 50 years) displaying invasive recurrence-free survival (IBCR) according to HER2 status of the primary DCIS among young patients (≤50 years at diagnosis), (**c**) and older patients with age 50 years or above at diagnosis (**d**)

The survival analyses were stratified for ER-status, and for patients with an ER negative DCIS, HER2 positivity predicted a significantly lower risk of IBCR (Log-Rank, *P* = 0.003), (Fig. 3a), which was not the case for ER positive DCIS patients (Log-Rank, *P* = 0.76), (Fig. 3b). In analyses restricted to women who undergoing BCS, ER status remained an effect modifier for the association between HER2 status and IBCR.

The prognostic value of age was studied in survival analysis using age as at dichotomous variable of ≤ 50 years (*N* = 141, 31 %) or > 50 years (*N* = 317, 69 %). Age was not proven to be a prognostic indicator of neither invasive nor *in situ* recurrence (data not shown). However, age added substantial information to the prognostic value of HER2 status. For presumably premenopausal women aged 50 or below, HER2 expression in the DCIS revealed no prognostic information (Fig. 3c). In contrast, for women who were older than 50 years of age at diagnosis, HER2 positivity was associated with a markedly improved recurrence-free survival for invasive disease (Log-Rank, *P* = 0.002), (Fig. 3d). The age-stratified analyses for *in situ* IBEs did not reveal any significant results.

## Discussion

In this long-term follow-up DCIS cohort, positive HER2 status in the primary lesion predicted lower risk of late invasive breast cancer recurrence compared to negative HER2 status in the primary DCIS. HER2 positivity did on the other hand indicate a non-significant higher risk of local *in situ* IBEs.

DCIS is by definition a non-invasive disease exerting a rather high risk of local recurrence but an especially low risk of lethal outcome from the disease [27]. However, an increasing rate of early-stage disease, including DCIS, is observed due to mammography screening [28]. The ideal screening method aims at fractioning indolent disease from cancers that will ultimately cause harm if undetected at an early stage. Current mammography methods are incapable of characterising the aggressiveness of a given breast tumor, and tissue based assessment of biological entities is warranted for improved clinical decision-making [1, 29, 30]. DCIS includes a plethora of diseases ranging from conditions where active surveillance might be adequate, to conditions where tumors should be surgically removed followed by adjuvant treatment. Prediction of recurrences is thus a high priority; both prognostic and treatment predictive factors should be identified [29].

Previous studies have shown increased risk of DCIS recurrences among patients with HER2 positive disease in the primary DCIS [31, 32]. These findings were vaguely indicated but not statistically significantly confirmed in this study. More importantly, HER2 status was predictive of invasive recurrences in this study showing that HER2 positive tumors were less reluctant to recur as an invasive tumor. The positive prognostic value of HER2 in DCIS demonstrated here might seem counterintuitive given the data showing HER2 to be positively related to tumor size, multifocality and nuclear grade and may reflect an independent role of HER2 in DCIS as a long-term prognostic biomarker. This study includes an extensive long-term follow-up, which enabled the detection of the lower incidence of late-recurrences among patients with HER2 positive DCIS. The Kaplan-Meier curves for invasive recurrences were concordant for several years, and not until 10 years after the primary DCIS, did the curves separate. The discordances between our results and the results generated by Kerlikowske *et al.* [32] may well be explained by the differences in follow-up time. More importantly, the endpoints applied in this study are not identical to previous studies among which any ipsilateral recurrence regardless of type has served as endpoint. Our results stating that HER2 positive DCIS is a beneficial prognostic marker for invasive recurrent disease highlights the need for studying several different endpoints to understand the prognostic value of a given biological marker. Presumably the key endpoint should be invasive recurrence representing the actual threat to the patient’s life as discussed in the review by Benson *et al.* stating the main goals of DCIS treatment to be prevention of recurrent invasive disease, minimised treatment-related morbidity, and optimised cosmetics [29].

In a previous publication, we evaluated the prognostic impact of molecular subtypes defined by the immunohistochemical surrogate classification [24]. The subtypes were based on the proposed classification for invasive breast cancer according to the St Gallen consensus meeting in 2011 [20]. HER2 cases were thus presented in two subtypes, i.e. Luminal B(HER2+) and HER2 + (ER-/PR-) leaving few cases in each subtype, which might explain the non-significant data generated in that study. Herein, we report significant results for HER2 as a prognostic marker for recurrent invasive disease, presumable prone to the facts that, in this study, all HER2 positive DCIS were analysed together and moreover, the follow-up period has been extended.

The significantly reduced risk estimates for patients with HER2 positive DCIS might help identifying a low-risk group for whom adjuvant treatment after surgical excision could safely be omitted. To keep overtreatment to an innocuous minimum is essential for DCIS-patients among whom a substantial amount of the lesions may be of limited clinical importance [28].

This study has several strengths, such as its relatively large sample size and confirmation of all events by a physician. Another important strength was the long-term follow-up of more than 15 years (mean). During follow-up invasive recurrences were identified in 23 % (106/458) of the women with a previous DCIS. From a biological point of view, the identified regional or distant metastases have most likely evolved from an invasive foci in the breast, which could have occurred at any time between the DCIS diagnosis and the diagnosis of metastatic disease, although not detected and therefore not possible to report in this study. The number of invasive recurrences does, however, correspond with numbers from previous publications on DCIS patients showing a recurrence rate of 25 % within 10 years of follow-up, 50 % of these being invasive recurrences [33], and 18 % during 5 years of follow-up [34]. Unequal recurrence-estimates may be explained be differences in follow-up length and the fact that no systemic endocrine treatment is recommended Swedish DCIS patients.

Some considerations are worth discussing for the interpretation of our results. All evaluated tumor biomarkers were investigated on TMAs, which may have limited the assessment of heterogeneous expression compared to whole sections. Previous validation studies of TMA-based biomarker assessment, however, show accurate concordance between TMAs and whole sections motivating use of TMAs for routine breast biomarker (ER, PR, HER2) analyses in the clinical setting [35]. A previous publication including a subset of this study population, showed an overall concordance of 80 % between TMAs and whole sections for IHC assessment of ER, PR, and HER2, and notably the prognostic value of these markers was similar irrespective of assessment based on TMA or whole sections [36]. This study is predominantly based on SISH regarding HER2 status with an anticipated high accuracy compared to IHC [37]. Breast conserving surgery represents the surgical intervention predominantly used in our cohort. Compared to mastectomy, the outcome following this surgical approach requires higher accuracy in pre-surgical imaging of the tumor extend. No data on pre-surgical imaging was however available for this study and differences could not be addressed further, The challenge of surgical margins differ depending of surgical method as evidenced in this cohort with clear margins obtained in 94 % of cases undergoing mastectomy compared to 86 % among the women who underwent breast conserving surgery. However, no association between surgical method and HER2 status was found, and the risk of confounding by surgical method is considered small.

HER2 positive disease is a strong predictor for impaired recurrence-free survival in invasive breast cancer, opposing the results on DCIS in this study. This controversy may reflect the diverse expression of HER2 in invasive lesions and adjacent DCIS components as recently discussed in a review by Cowell *et al.* [30]. The tentative explanations given are that HER2 amplification may either have been lost in the progression from ductal carcinoma *in situ* to invasive breast cancer, or that the invasive component has arisen from a DCIS clone without HER2 amplification [30]. Biologically, the striking issue is why progression to invasive disease would imply loss of HER2 amplification, which warrants further attention.

Although this study implies, that patients with HER2 positive DCIS are less likely to experience recurrent invasive disease, the HER2 positive DCIS disease has substantial clinical interest as a targetable early-stage disease. This has been acknowledged by recently conducted clinical trials testing the efficacy of HER2 targeting therapy with lapatinib in DCIS patients (ClinicalTrials.gov NCT00555152 and NCT00857714, respectively). The results of these trials are currently not published, however the trial results by Estevés *et al.* assessing lapatinib in the pre-surgical setting reported significant inhibition of HER2 signalling and reduced tumor size following 4 weeks of treatment in HER2 positive DCIS patients [38]. Further, other biomarker trials have assessed the effects of trastuzumab in DCIS patients, among which, one trial has reported the results, showing no significant tumor effects in terms of proliferation and apoptosis after a single-dose monotherapy trastuzumab pre-surgically [39]. None of these studies have addressed the long-term benefits of HER2 targeted therapy in DCIS. However, a currently recruiting phase III randomized trial prescribing trastuzumab versus placebo concomitantly with radiotherapy, may shed light over the long-term effects (ClinicalTrials NCT00769379).

## Conclusions

Clinical decision making for DCIS patients is still challenged by the inability to predict recurrence or, most importantly, progression to invasive disease. In line with the models developed for invasive breast cancer, such as Adjuvant! [40], a comprehensive set of solid biomarkers for DCIS prognosis and treatment prediction has repeatedly been requested. In this large DCIS cohort with extensive follow-up, we demonstrate significantly improved long-term invasive disease-free survival for patients with HER2 positive disease in the primary DCIS. These results indicate that HER2 status could add significant information in future development of prognostic and predictive DCIS models.

## Abbreviations

BCS: Breast conserving surgery
CI: Confidence interval
DCIS: Ductal carcinoma *in situ* of the breast
ER: Estrogen receptor
HER2: Human epidermal growth factor receptor 2
HR: Hazard ratio
IHC: Immunohistochemical
IBCR: Invasive breast cancer recurrences (invasive ipsilateral/regional/contralateral recurrence or distant metastasis)
IBE: Ipsilateral breast cancer event
PR: Progesterone receptor
RT: Postoperative radiotherapy
SISH: Silver-enhanced *in situ* hybridization
TMA: Tissue microarray
